# Co-evolution of groups and opinions in an agent-based model

**DOI:** 10.1371/journal.pone.0338486

**Published:** 2025-12-12

**Authors:** Duncan Cassells, Antoine Vendeville, Lionel Tabourier, Pedro Ramaciotti

**Affiliations:** 1 LIP6, Sorbonne Université, Paris, France; 2 Médialab, Sciences Po, Paris, France; 3 LPI, Learning Transitions, CY Cergy Paris University, Paris, France; 4 Complex Systems Institute of Paris Île-de-France, CNRS, Paris, France; University of Hamburg: Universitat Hamburg, GERMANY

## Abstract

This study explores a model for the co-evolution of opinions and groups, related to opinion polarization and group identity in opinion dynamics. While traditional models focus on pairwise interactions, we incorporate the notion of groups thereby allowing agents to identify other agents as in-group or out-group. By modifying key parameters we examine how understanding interactions as in-group or out-group affects the convergence or divergence of opinions. Our findings reveal that attitude towards out-group plays a leading role in the polarization of such systems, while in-group interactions can temper extreme opinion shifts or even fragment groups. This model offers an understanding of the complex interplay between group identification and polarization, providing a contribution to ongoing debates about segregation and sectarianism in public and private spheres.

## Introduction

In recent years, there has been increasing discussion among social scientists [1–7] as well as computer scientists and specialists of complex systems [8–13] of the causes and effects of polarization among the public, and the resultant impact on opinions and attitudes. Among the various approaches for studying these phenomena, opinion dynamics models and simulations provide frameworks to explore the role of diverse mechanisms in the emergence and evolution of polarization. We refer the interested reader to [14–16] for extensive reviews of the field of opinion dynamics.

The vast majority of opinion dynamics models are based on pairwise influences or exposures, whereby agents repeatedly update their opinions after one-on-one exchanges. The notion of group influences has been more recently studied through higher order networks [17] and threshold effects [18–21]. These works consider, however, dynamics at a widened local level rather than the role of *group identification*. That is, the idea that individuals feel a sense of belonging to a perceived group, and shape their influence with others depending on whether or not they are recognized as belonging to the same group [22].

One of the most concrete examples of group identity is the identification to a political party. This frame of analysis is particularly salient in the United States, where the reinforcement of party identification is posited as a driving force of opinion polarization, aversion towards others, and overall deepening political conflict [1,23,24]. Political party identification is also a good predictor of positions on several policy issues [25], as parties and their partisans exhibit increasing ideological alignment [24]. In online social platforms, users are preferentially exposed to like-minded content (the so-called filter bubbles or echo chambers; see [13,26–28]), while communication tends to be mostly hostile between different camps [29–31]. Overall, group identification plays an important role in opinion communication, formation and ultimately in political polarization [32,33]. Therefore, it is crucial to develop this notion in the context of opinion dynamics models.

The importance – as well as the complexity – of group dynamics in online polarization has recently received renewed attention in relation to proposed interventions. One stream of research pointed to diversification of content and bridging between political groups as tools for moderating potentially exacerbated polarization [34,35]. However recent results have shown that certain cross-cutting exposure of content and arguments between groups can further entrench and even radicalize positions, essentially backfiring [36,37]. Some works have connected out-group contact with the ability to challenge held views [38] and even fostering compromise [39], while others have stressed opposite effects linked to exacerbated polarization because individuals are prone to motivated reasoning, thus increasing the perceived differences in stances between out-groups [40]. This puzzle has in turn motivated the development of opinion dynamics models capable of accommodating these empirical findings, via the inclusion of repulsion mechanisms [19–21,41–43]. The Attraction-Repulsion Model (ARM) [44] is an agent-based model that hinges on the perception, on the part of agents, of a binary distinction: opinion updates arising from the influence exerted between agents depend on the relative opinion distance, and whether this distance exceeds a threshold. This distinction allows to describe the combined effect of filter bubbles and backfire effects. This model falls into the well-known category of bounded confidence models [45–47], where agents are drawn towards each other when they hold close enough opinions, and do not interact otherwise, but extends it by allowing for agents to be repelled (instead of drawn to each other), depending on how distant they are in the opinion space.

In this paper, we build on this binary distinction present in the ARM and further formalize the integration of the notion of groups, as opposed to considering only a threshold for distance as the means by which agents can discriminate in processing the opinions of others. We incorporate a group identification process into the dynamics, through which agents infer agroupments in the population [48]. Agents are then exposed to and influenced by others depending on whether they belong to the same group (*in-groups*) or not (*out-groups*). We investigate the consequences of this distinction on the level of opinion polarization in the population, as evaluated by the Duclos-Esteban-Ray measure [49]. A key element of our proposed model (in comparison to existing models) is that fragmented groups do not only emerge as a consequence of the opinion dynamics determined by the parameters that define pair-wise influence. In our model, groups are abstractions that are analytically available to the model, and that are treated as arguments for other parameters. Consideration of an out-group is shown to be the primary factor modulating the tendency of the system of agents to converge to polarized states. A key parameter of the ARM affected by perception of in- and out-groups is that of the *tolerance* of an agent, meaning the distance in opinion space beyond which the influence turns from attractive to repulsive. In our model, groups are formal attributes of agents, on which model parameters can be predicated as functions: e.g., we explore simulations in which *tolerance* in ARM is dependent on whether the influence an agents exerts on another happens *within* or *across* groups. A central finding of our work is that lower tolerance to out-groups, by contrast with tolerance of distant opinions among in-groups, is the main driver of group polarization and consequent divergence in opinions within the population. The distinction between in-groups and out-groups in the other models parameters, *exposure* and *responsiveness*, also impacts polarization, although to a lesser extent.

The treatment that agents undergo in our model holds resemblance with the affective treatment of others at the core of *affective polarization*, understood as the prevalence of distrust and hostility between members of different groups (e.g., supporters of different political parties). The research questions that arise from the inclusion of group identification are: a) at what point does differentiating treatment of in-group and out-group affect the evolution toward polarization or consensus, b) are the parameters determining in-groups or out-groups more important for understanding polarization than those determining influences between two agents, and c) how does the shift from individual to group focus change the framing of existing opinion dynamics work? Given the importance granted to affective polarization in the literature, we move away from the view of agents as identical towards a more nuanced description of how individuals treat each other. Our model addresses, however, more broadly the modeling of group structures in systems in which agents exert pair-wise influence. We provide in the discussion a set of additional features that may increase the realism of our model in accounting for its application to political opinion polarization.

The article is organized as follows. We first describe the opinion dynamics model and the effect of group perception and identification therein. Then, we detail the mechanism by which agents perceive and identify the different groups. Then, we proceed to specify a scope of simulations that explore the effects of group perception and identification in the ARM. Finally, we present the results of these simulations, unfolding the consequences of the aforementioned modeling choices and scope of parameters considered in simulations, followed by concluding remarks.

## Model description

Prescribing a model for our purposes includes setting out 1) an exposure rule, 2) an influence (or opinion update) rule for pairwise influences, and, crucially, 3) a group perception and identification rule.

The model consists of a population of *N* agents, each having an opinion $\sigma_{u}$, with u∈1,…,N, who interact with each other and change their opinions as a result of these influences and their group identities. The opinion is a value on a continuous scale between 0 and 1—this may be conceptualized, for example, as a Left-Right scale in a real world scenario. The opinion difference $d_{uv}$ between actors *u* and *v* is defined as the distance between their opinions, that is $\sigma_{v}-\sigma_{u}$. In the following subsections we present rules for influence, opinion update, and group identification, which govern how the population’s opinions evolve.

Because this model builds on the ARM by [44], it includes the three parameters originally proposed: 1) tolerance (a threshold for $d_{uv}$ beyond which attraction switches to repulsion), 2) responsiveness (the amount of attraction/repulsion exerted by an agent over another), and 3) exposure (modulating the probability of two agents influencing each other depending on how distant they are in opinion space). The rest of this section describes how to integrate group perception and identification, allowing these three parameters to be dependent on whether a pair of agents fall within or outside of a social group.

### Exposure rule

When observing and interacting with opinions there is a possibility of exposure to distant opinions (for example, through chance encounters, family connections, workplaces) and those opinions which are more similar to the observer. As such influences should be possible between all agents, but the likelihood of an agent being exposed to the opinion of another should reflect the principle of homophily. That is to say, individuals are more likely to interact with those that they are similar to, widely acknowledged as a determinant of exposure in the literature [50]. Therefore agents in the model have a non-zero probability of exposure with any other agent in the population, however exposures become increasingly likely between those who are more similar in opinion (a smaller absolute opinion difference $\left|d_{uv}\right|$).

To reflect this, we have a complete directed network (so any agent may be exposed to any other agent’s opinion) with weighted edges between agents (the likelihood that an exposure occurs). The likelihood of exposure is a function of the opinion difference between actors and an *exposure* parameter, *E*. Our model comprises discrete time steps, and an independent random exposure network computed on each time step. The probability that an actor *u* observes the opinion of *v* at any given time step is

$$P(u\to v)=(1/2{)}^{|d_{uv}|/E}.$$ (1)

**Fig 1:**
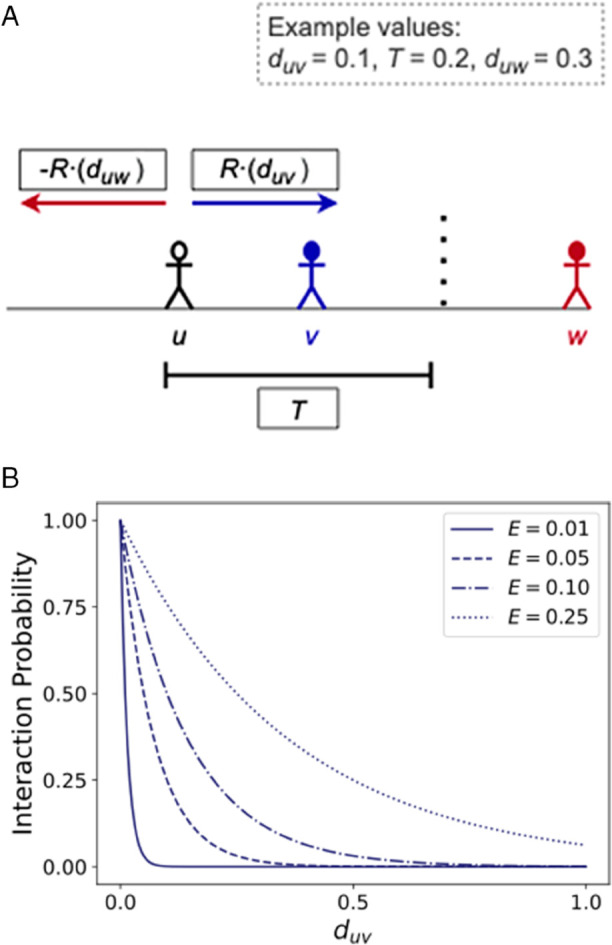
(a) Exposure between agents *u* and *v* is attractive since $d_{uv}$ is less than prescribed tolerance *T*, while influence between *u* and *w* is repulsive since *d*_*uw*_ is greater than the tolerance threshold *T*. (b) The probability that two agents interact given their opinion difference, under different levels of exposure.

This prescribed likelihood function reflects an increasing likelihood of exposure as opinions are more similar, while *E* plays the role of a shape parameter. Some illustrative likelihood functions for different parameters may be seen in Fig 1 to get a sense of how *E* impacts the chance of interacting with agents of significantly different opinions. To create the directed exposure networks, at each step we compute the probability of each one of the *N*^2^ potential edges existing in the system, and we draw them as Bernoulli random variables.

### Opinion updating rule

Following an exposure, an agent will update their opinion. This will either be an attractive or repulsive update; that is, the agent’s opinion will become more or less similar to the observed opinion by reducing or increasing opinion difference on the opinion scale. This reflects the desired behavior that agents distance themselves from significantly different opinions while assimilating with similar ones.

We employ two model parameters: *tolerance*, *T*, and *responsiveness*, *R*. Tolerance is the opinion difference at which influences from exposure flip from being attractive ($|d_{uv}|\leq T$) to repulsive ($|d_{uv}|>T$). Responsiveness is the fraction of the opinion difference by which the updating actor will change their opinion by — one extreme is to completely adopt the new observed opinion, the other is to not change opinion at all. With this we can express the opinion update rule for agent *u* at a time step *t* resulting from an exposure to the position of agent *v* as

$$\sigma_{u}(t+1)=\sigma_{u}(t)+\text{sign}(T-|d_{uv}(t)|)\times R\times d_{uv}(t).$$ (2)

Notice that opinion difference is calculated in the direction from *u* to *v* to determine the correct sense for attraction/repulsion.

Fig 1 illustrates a situation with three agents *u*, *v*, and *w*. Agent *u* interacting with *v* is attractive, while *u* interacting with *w* results in repulsion given that the opinion difference is greater than the tolerance threshold. The adjacent figure indicates the likelihood of exposure, as parameter *E* decreases or opinion difference increases the probability becomes less likely.

### Group identification

The final part of the model introduces group perception and identification. We want to reflect that agents treat their own in-group differently to the out-group, which results in different levels of values *E*, *R*, and *T*. Therefore in our model we take each of the three parameters, tolerance *T*, responsiveness *R*, and exposure *E*, and split them into an in-group parameter and an out-group parameter one-by-one. We then organize our simulations into three groups, independently considering in- and out-group tolerance (keeping a common responsiveness and exposure; i.e., $\left\{T_{\text{in}},T_{\text{out}},R,E\right\}$), responsiveness (keeping a common tolerance and exposure; i.e., $\left\{T,R_{\text{in}},R_{\text{out}},E\right\}$), and finally exposure (keeping a common tolerance and responsiveness $\left\{T,R,E_{\text{in}},E_{\text{out}}\right\}$). This will allow us to then dissect the differing roles of in-group and out-group exposure, responsiveness, and tolerance in the opinion model.

Introducing group-dependency on top of the variables in the ARM implies a potential connection between social identity and these variables. At the simplest level, the existence of in-group favouritism and out-group discrimination [33] may be applied to each variable that operates intra-group and inter-group as these biases could be present. More specifically however, there is evidence that assimilation of content can be greatly increased by feeling of closeness to another person [51] and that people are more likely to act on the opinions of their in-group [52], both of which relate to group-dependent responsiveness. The negative relationship between positive evaluation of out-group arguments and positive identification of the other as out-group [53] points to the possibility of group-dependent tolerance. And finally, it has been seen that group-dependent exposure can exist, in part due to recommendation systems, in digital spaces [13,28,54] but also self-organization in physical space [55].

Group identification within our model is determined by the distribution of the opinions of the agents and similarities, or clustering, within it. In reality, however, a multitude of social and political dimensions contribute to group identity such as political parties, ethnic groups, professional groups, and so on [22]. For the purposes of this article we rely on a simple modeling of the group identification process, while acknowledging the existence of more complex underlying mechanisms. In our model, the attribute that is communicated in exposures, i.e., the opinions, is also the same attribute on which agents perceive agroupments and identify into perceived groups. Furthermore, our model does not accommodate means of accounting for possible persistence in the perceived group identity, as agents identify into groups purely on the basis of their current opinion. A concrete example of a context where these assumptions are violated, is provided by many political settings in Europe, where multiple opinion dimensions are necessary to distinguish political groups from each other [56]. Our model provides, we argue, the first tool to address co-evolution of groups and opinions, and we offer in the conclusion paths to enriching it in view of matching different empirical settings.

Persistence mechanisms are well known to the opinion dynamics literature regarding individual positional opinions. One traditional approach consists in explicitly including a parameter moderating the relative weight between new influences (acting on a given time step) and past positions held by the agent (e.g., [57]). A second popular approach is the use of self-influence: when the influence of agents *j* over agent *i* is computed, typically encoded in an influence matrix with elements *w*_*ij*_, the self-influence *w*_*ii*_ accommodates the relative weight between persistence of the previously held position (in the immediately past time step) and the influence of other agents (e.g., [58]).

The operationalization of the persistence of groups (i.e., the degree to which agents resist changes in groups), as they are formalized in our model, does not have immediate extensions starting from the individual position persistence mechanisms described. This lack of natural extension relates to group indices being categorical, as opposed to continuous positional quantities. We also remark that the persistence of groups that do not depend on the positional opinions of agents simulated in opinion dynamics models, might also be of importance to capture relevant groups in empirical settings (e.g., demographic groups). S3 Fig of the Supporting Information document, S1 File, provides an illustration of a simulation using our model, but fixing groups at the start.

### Comparison with existing opinion dynamics models

The model proposed in this section exhibits both similarities and differences relative to other opinion dynamics models. Two fundamental features of our model are derived from Axelrod’s Attraction-Repulsion Model (ARM [44]), upon which this work is predicated: (1) bias mechanisms and (2) distancing effects, modeled by the *E*,*R*,*T* parameters. Through the exposure parameter *E*, the ARM incorporates a bounded confidence effect by which agents preferentially interact with others who are sufficiently close in the opinion space, a recurring mechanism in opinion dynamics models since the seminal works of Axelrod (1997) [47], Deffuant et al. (2000) [46] and Helgselmann and Krause (2002) [45]. In these models, agents interact only if their opinions differ by less than a fixed threshold, while in the ARM, the confidence bound is a continuous function of opinion distance, allowing for interactions far across the ideological spectrum—albeit with low probability. At the same time, the ARM integrates distancing effects (modelled by the tolerance parameter *T*) by which agents are repelled away from those holding discordant opinions. These distancing effects have been documented in the literature since (at least) the early 2000s [59,60]. Finally, the responsiveness parameter *R* allows us to modulate the extent to which agents are willing to reassess their opinions after interactions, reminiscent of opinion persistence mechanisms in other models [58].

The primary distinction between the proposed model and existing approaches lies in the manner through which groups are imposed and operationalized within the computational rules governing opinion updates at each temporal iteration. Whereas in most models groups manifest as emergent macro-properties, the present framework treats group identification as a prescribed computational element at each step, thereby rendering group-based rules explicitly specifiable. For example, many models (encompassing the bounded confidence models mentioned above) take interest in the study of how pair-wise opinion dynamics may induce the fragmentation of the population into ideologically homogeneous clusters. In contrast, in our model, group identification and attribution for agents constitutes a variable that serves as an argument within model parameters conceptualized as functions. Group indices for each agent are categorical values that are arguments of model parameters: responsiveness *R*, tolerance *T*, and exposure *E*. This functionality enables the modeling of differential tolerance values for in-group versus out-group exposures, operationalizing social psychological principles that motivated the ARM proposition [44] and addressing recent empirical observations [36].

## Grouping mechanism

We now turn to the modeling of the mechanisms through which agents identify groups in social systems, on the basis of opinions. This choice is motivated by the literature on group boundaries, identified by actors along the lines of symbols such as displayed behaviors or opinions (e.g., symbolic boundaries in sociology [32]). Following this theoretical perspective, our rationale for putting forward mechanisms through which agents might perceive and identify groups in the system relies on the the degree to which they can cluster other agents on the basis of their positions on opinion space.

To do so we rely on existing clustering methods dependent on the shape and density of the opinion distribution, and infer how actors may perceive their resulting group membership. Our defined group identification is then a function of the opinion position of each member of the population. We note that tolerance and group identity are closely linked, although not the same, since both make use of opinion difference. As an example of the difference between tolerance and group identity, if agents *u* and *v* are part of the same group but at either edges of the spread of the group’s opinions, then it is possible that they are sufficiently far apart that $d_{uv}$ is greater than *T* despite being members of the same group. At the same time, another agent *w* might be part of a different group but below the tolerance threshold in opinion difference to *v*.

We use the HDBScan clustering method [61] over the set of opinion positions on the whole population as an operator yielding a cluster apportionment in the system. We set a minimum group size of *N*/5 and run the procedure at each iteration; i.e., at each time step, agents re-evaluate the group composition of the population. However, our model can accommodate various group-identification procedures (extending beyond the HDBScan methodology employed in the present study) and imposes no constraints on the number of groups. Consequently, a grouping procedure may yield anywhere from 1 to *N* distinct groups. In the limiting case where only one group exists, the model becomes functionally equivalent to the ARM, wherein all exposures are classified as in-group influences, thereby yielding an ARM specification with $T=T_{\text{in}}$, $R=R_{\text{in}}$, and $E=E_{\text{in}}$. Conversely, when each agent constitutes its own group, the model again reduces to the ARM framework, wherein all exposures are categorized as out-group influences, resulting in an ARM specification with $T=T_{\text{out}}$, $R=R_{\text{out}}$, and $E=E_{\text{out}}$. Re-evaluation of groups at every time step may not reflect the ‘stickiness’ of some elements of group identification, but we find it to be valid in this context since groups in this article are defined on top of the opinions so if the opinion distribution changes then the group assignments must also reflect this. If group identification were to be static (or ‘sticky’) over time then the model would not be able to assess behaviors such as group fragmentation or joining. For example, if two groups in the population come to consensus by sharing the same opinion this should now be one group rather than reflecting the previous two groups, or if a small lot of agents splinters from the group then this should be recognised. In practice, the lack of new identified splinter groups can mean that the middle of the opinion space is less likely to be populated and results in higher polarization (outcome shown in the Supporting information, S3 Fig). It should be noted that under this apportionment, all agents concur on the number and the composition of social groups. Possible alternatives might include agent-specific or subjective perception of social groups. While this would be a valid modeling choice that might find motivation in social theory, we deliberately choose the simplification to introduce the analysis of group effects in this work. When actors do not clearly belong to a designated group (an option accommodated within the HDBScan clustering algorithm), they are classified as ‘No-Group’ and subsequently treated as constituting a collective group within the system. Specifically, exposure rules are configured according to in-group parameters, with influences determined based on in-group tolerance and responsiveness specifications. The theoretical rationale underlying this modeling decision is to account for scenarios wherein such agents recognize other non-group members as fellow outliers, thereby forming a cohesive identity based on their shared peripheral status. The alternative approach—treating each ‘No-Group’ agent as an individual group—would model situations potentially relevant for certain empirical contexts but exerts no substantive impact within the scope of the present study. The results presented herein pertain to simulations employing bimodal distributions to examine the interplay between affective and ideological polarization. For further details on the implementation of the HDBScan algorithm, see [62].

**Fig 2:**
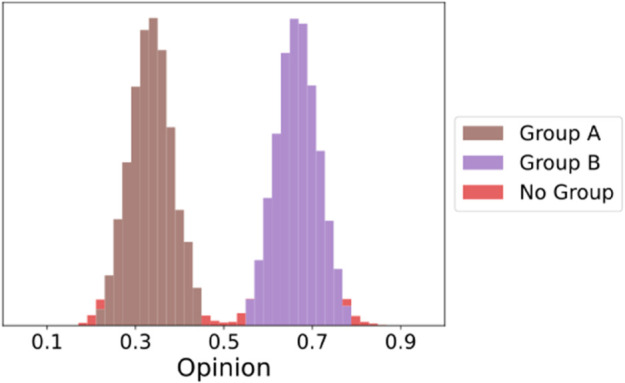
An example assignment of group identification using clustering on a typical initial distribution of the population. Two groups are identified and edge cases are assigned no group.

The benefits of this approach are that it does not require a predefined number of groups, and individuals may be left not belonging to any group. Fig 2 displays the resulting groups of a population drawn from a Gaussian mixture model with two distinct normal distributions for components. Applying the clustering operation on this distribution successfully retrieves two groups and group extremity points are left as “No Group”, showing that they are outliers. Furthermore, the process is fast enough and does not extend computational time unreasonably, with an 𝒪(N·log(N)) complexity [63].

## Results

### Experiment protocol

We organize our simulations as follows. The number of agents is set to *N* = 100. At the top level, simulations are organized into three types, according to what parameters (*T*,*R* or *E*) will be separated into in- and out-groups, resulting in three main sets of simulations. For the parameters that are not split into in- and out-groups, we explore three values that exhibit different behaviors, typically two extreme values to demonstrate the range of the system’s behavior and one intermediate value that shows multiple possible outcomes when varying the group-dependent parameter. Precise values are determined by the context of the group-dependent parameter and detailed below. For the parameter that is split into in- and out-groups, we explore 20 values between 0 and 1. This yields the following sets of parameter exploration in the three top level types of simulations:

- Group-dependent tolerance (GDT) simulations:
  $T_{\text{in}}\in [0,1],T_{\text{out}}\in [0,1],R\in \{0.01,0.1,0.25\},E\in \{0.01,0.1,0.25\}$;
- Group-dependent responsiveness (GDR) simulations:
  $T\in \{0.01,0.1,0.4\},R_{\text{in}}\in [0,1],R_{\text{out}}\in [0,1],E\in \{0.01,0.05,0.25\}$;
- Group-dependent exposure (GDE) simulations:
  $T\in \{0.01,0.1,0.4\},R\in \{0.01,0.05,0.25\},E_{\text{in}}\in [0,1],E_{\text{out}}\in [0,1]$.

For a given set of parameters *T*, *R* and *E*, regardless of which is split into in- and out-group parameters, we run a simulation 20 times, and report on the average outcomes. Regarding the number of runs adopted, the literature knows a broad range of values in a trade-off between managing computational complexity and achieving statistical significance. Studies on simulations computing costly LLM outputs on each iteration have reported as little as three runs in exploration for sensitivity and stability [64]. The number of runs averaged when reporting average system properties knows examples ranging from tens (e.g., in similar settings in the 2002 Hegselman-Krause model [45]), to hundreds [65] and thousands [66]. Because some of our simulations run 1000 time steps, we average over 20 runs for each set of parameters to manage the computational complexity of our study.

Each time we instantiate the simulation drawing half of the opinions (50 agents) from a Normal distribution $𝒩(\mu_{1},\sigma_{1}^{2})$, and the other half from a Normal distribution $𝒩(\mu_{2},\sigma_{2}^{2})$, with $\mu_{1}=1/3,\mu_{2}=2/3,\sigma_{1}=\sigma_{2}=0.05$. The resultant starting distribution of opinions is always in two groups and resembles those used to demonstrate the grouping mechanism in Fig 2. Initialising the experiments with two groups is of particular interest in our context since we want to understand group behaviors—with a unimodal or uniform distribution we would begin with one group or no group, respectively, both of which are not the focus of this article which studies in-/out-group exposure. Some results with different starting distributions can be found in the Supporting Information. For each set of parameters and for each drawn population at the first time step, we let the simulation run until convergence of the measured polarization of the population.

Polarization of the opinion distribution at each time step is given by the Duclos-Esteban-Ray (DER) measure [49], which allows us to compare across subsequent opinion distributions with a single value of polarization for each. The exact formula is

$$P_{\alpha }(f)\equiv \int \int f(x{)}^{1+\alpha }f(y)|y-x|dydx;$$ (3)

where *x* is an opinion, *f*(*x*) is the density at *x*, and we set α=0.5. For implementing the measure we use kernel density estimation to approximate *f*(*x*) and the sample based estimator for $P_{\alpha }(f)$ described in [49, Sect 4]. The starting value is typically 0.21—the exact value varies depending on the precise random initialisation of opinions —which is mild polarization, between the minimum value 0 and maximum value 0.5.

The DER measure is thus constituted of a parametric family of functions, designed to accommodate desired properties of polarization measures. Axiom 1 posits that, in uni-modal distributions, polarization increases with spread. Axiom 2 posits that, if a distribution is a mixture model, a decrease of the spread of each component of the mixture increases polarization. Axiom 3 posits that, if a distribution is a mixture model, when components of the mixture model are distanced further apart, polarization increases. Axiom 4 imposes scalability and order for the measure. In other words, the parametric DER family of measures acting on a bimodal distribution is defined such that polarization increases if modes grow further apart, or if the distribution of each mode is further concentrated around the means of the modes. S4 Fig of S3 Sect of the Supporting information provides an illustration of different DER values for a bimodal 2-component Gaussian mixture model with different separations between components and spreads.

Each simulation is run for up to one thousand iterations, or terminated before that if opinions have not changed over one hundred consecutive iterations (X = 100 in Algorithm 1). Convergence is discussed in Supporting information S2, and curves of the DER value approaching convergence are displayed in S2 Fig. At each time step, each agent evaluates first the group composition of the system, then whether it is exposed to every other agent following the exposure rule, and finally updates its opinion based on the opinion update rule. Note that all updates are synchronous, so agents *i* and *j* update their opinion at the same time following the exposures. An outline of the full experiment process is presented in Algorithm 1. The results that we report in the next pages for each parameter set is the average of the final one hundred time step iterations across the twenty experiments. While the numerical simulations may evolve in up to a thousand time steps, our convergence criterion allows us to take the last hundred values to present the mean value.

**Algorithm 1 Each experiment for some set of parameters follows these steps.**

$\sigma_{1,\ldots ,N/2},\;\sigma_{(N/2+1),\ldots ,N}\leftarrow \;∼𝒩(\mu_{1},\sigma_{1}^{2}),\;𝒩(\mu_{2},\sigma_{2}^{2})$

iterations←0

**while** iterations<iterations_limit **do**

**for** $\sigma_{i}$ in σ **do**

**ExposureRule**($\sigma_{i}$)

**for** $\sigma_{i}$ in σ **do**

**UpdateRule**($\sigma_{i}$)

assign σ updated opinion synchronously

iterations+=1

**if** $\sigma_{1,\ldots ,N}$ unchanged over the X previous iterations **then**

break

### Types of polarization achievable by the model

**Fig 3:**
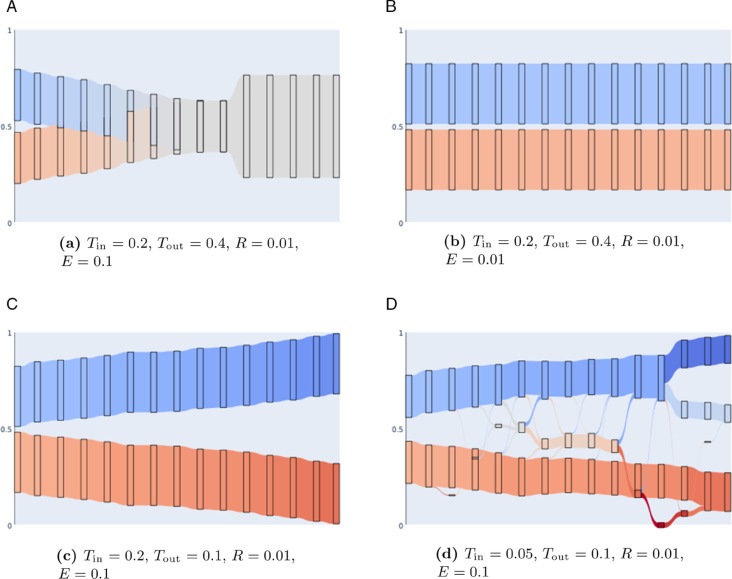
Illustration of qualitative types of polarization achievable by the model, displaying positions of agents in each of the two initial groups, and their evolution for the first fifteen time steps of a simulation. Vertical position is equivalent to the opinion position; i.e., position between 0 and 1 in opinion space. Groups are rectangular areas centered at the opinion mean of the group, with length representing the size of the group (not the opinion area they cover) hence a longer rectangle when the whole population is in one group (as in Fig 3a). Colour reflects the opinion value too. Four qualitative polarization types are illustrated: (a) consensus, (b) stable polarization, (c) unstable polarization due to group drift, and (d) unstable polarization due to group fragmentation.

Before exploring the parameter space of the model, we discuss typical dynamical behaviors that are exhibited during the simulations. Convergence to consensus and drift to a complete polarization are known behaviors in the literature, which have been described in many works on opinion dynamics. Because of the split of parameters into in- and out-groups, our model can also display other dynamical patterns that can be best understood with simple examples, such as the ones illustrated with alluvial plots (see Fig 3). S3 Fig of the Supporting information illustrates the behavior of similar simulations, in which groups are fixed and not dependent on evolving positional opinions.

To achieve consensus, actors in a group must be exposed to those in another group (therefore of differing opinion) and be sufficiently tolerant so as to be attracted towards their opinion. Over a series of iterations we will eventually arrive at some opinion shared by the whole population. Fig 3a illustrates such a situation in which the two starting groups gradually arrive at a consensus opinion and combine to become one group.

Stable polarization occurs when out-group exposure is so low that the out-group opinions are rarely seen, and when they are observed they are outweighed by the in-group opinion. Thus the two groups neither attract nor repel. In this case the groups remain polarized but not to the extremes of the opinion space since each group is in its own bubble (Fig 3b).

Our model can also achieve unstable polarization states. These are either intermediate polarization states that will eventually converge to stable polarized states, or, crucially, states that are unstable because of group fragmentation. We show this through two qualitative examples. The first occurs when a group is exposed to another but does not tolerate the difference in opinion, so actors in this group *drift* to the extremes by repulsion — an example of this can be seen in Fig 3c. Fig 3d illustrates a second and different situation, in which small groups of agents float in the space between the two groups due to fragmentation. The in-between agents then either change group, rejoin the previous group, or stay in the middle, during the next iteration. Maximum polarization is never quite achieved with the middle ground populated by some agents despite the two starting groups being repelled by each other.

### Effects of Group-Dependent Tolerance (GDT Simulations)

**Fig 4:**
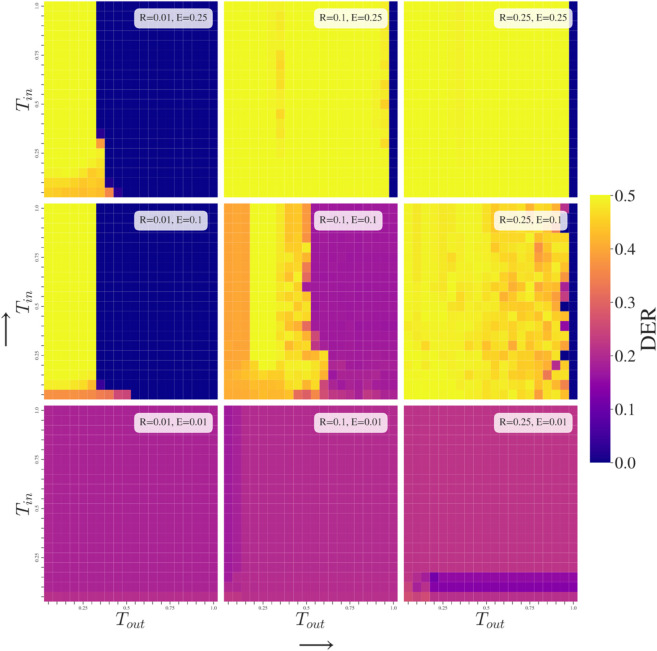
Group-Dependent Tolerance (GDT) simulations. The most important factor in determining polarization outcomes of the population is $T_{\text{out}}$ as it decides whether two groups will be attracted to each other or separate. This is dependent on *E* being high enough that out-group opinions are observed often enough to impact the in-group.

Regarding GDT simulations, Fig 4, we observe that at fixed *R*, higher exposure tends to increase the $T_{\text{out}}$ threshold at which we can have low polarization. While at fixed *E*, higher responsiveness results in more polarization as agents quickly find the extremes of the opinion scale from where it is hard to be attracted to any out-group opinion unless $T_{\text{out}}$ = 1. This aligns with the main findings reported by [44] without groups. If *R* is low enough, then the boundary at which $T_{\text{out}}$ leads to a highly polarized state or a lowly polarized state becomes very sharp.

$T_{\text{in}}$ typically does not affect the general behavior (for common *E* and *R* values) unless it is so low that it causes a fragmentation of the in-group from within. This can be observed under low responsiveness (*R* = 0.01) and some level of out-group exposure (E=0.1,0.25) where we do not arrive at maximum polarization for $T_{\text{in}}$ = 0.05 due to the middle ground between the two extremes being occupied by some agents (as illustrated in Fig 3d). At higher *R*, the middle ground agents’ opinion changes so drastically that it arrives at the extremes in one iteration, so they break out of the middle ground.

These observations can be appraised in Fig 4, in which the primary conclusion is that out-group tolerance is the main driver of behavior change in our model. Ultimately, it governs whether or not two groups can come together to consensus by attraction to each others’ opinions. It is not necessary that opinion difference with the whole out-group is below the tolerance threshold but that a sufficient number of out-group agents are within the limit to result in net attraction between groups. As such, the distance between groups being more or less than the out-group tolerance is critical to the resultant behaviors.

**Fig 5:**
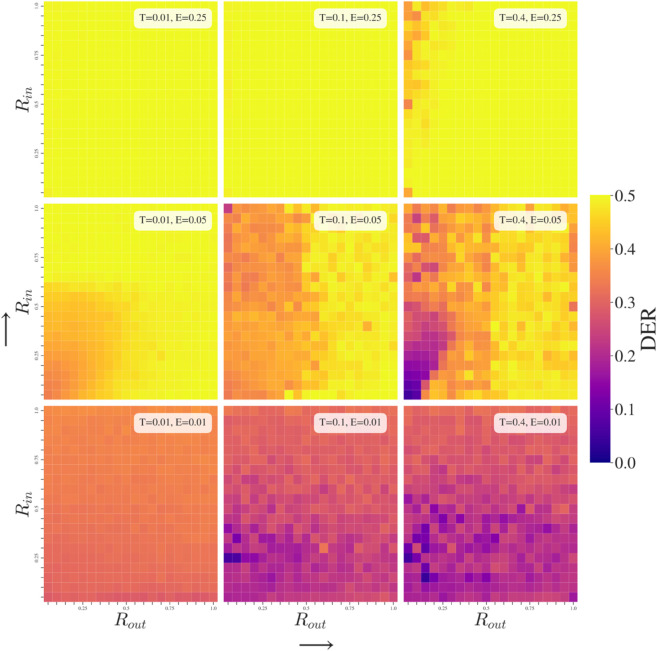
Group-Dependent Responsiveness (GDR) simulations. The size of response in attraction or repulsion governs a more gradual change of behavior when compared to the sharp boundaries for *T* in Fig 4. Out-group treatment is again most important when exposure to different opinions occurs regularly.

**Fig 6:**
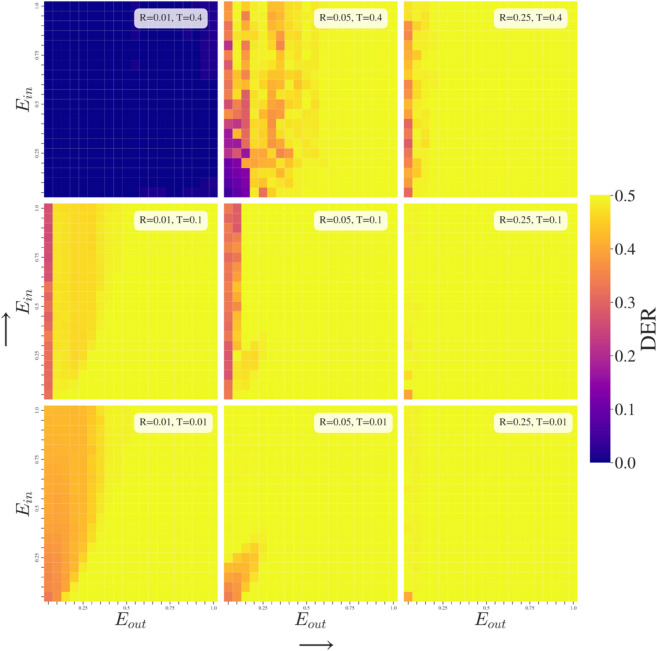
Group-Dependent Exposure (GDE) simulations. Over-exposure to in-group opinions and under-exposure to out-group is a scenario that mirrors concerns around filter bubbles. In our model, polarization is more likely with higher $E_{\text{out}}$ implying that polarization occurs from exposure to differing opinions that is not tempered by either high tolerance of opinions or larger exposure to in-group.

Where pixelation occurs in Figs 4, 5, and 6, it is due to volatility in the final DER polarization of the population across simulations, for which there are two reasons. The first is that fragmentation, as in Fig 3d, occurs and results in a variety of possible polarization values. The other is that the population has some probability of range of stable outcomes and so the average DER value across experiments is less predictable – for example, in Fig 4 when R=0.1,E=0.1, $T_{\text{in}}$=0.6, $T_{\text{out}}$ = 0.4, seventeen out of the twenty simulations result in a DER value over 0.4000 with the population split between two roughly equal groups at the extremes of the opinion space, however one simulation run ends (stably) with eighty-four agents of opinion 1 and the remaining sixteen agents of opinion 0 giving a resultant 0.2499 DER value, since one group is far larger than the other. This typically occurs at a boundary between zones of behavior or due to model parameter combinations that result in large opinion shifts of agents and so either end of the polarization spectrum may be achieved. The standard deviation of final state DER polarization across experiments reflects this variability and is therefore higher in areas where more pixelation occurs.

### Effects of Group-Dependent Responsiveness (GDR Simulations)

Responsiveness determines the intensity of the agents’ reactions in how they update opinions. When agents have low responsiveness to others they creep towards average opinions or slowly separate, while when the parameter is high they react strongly by pushing out to extremes of the opinion scale if repulsed or even overshooting attraction to a new opinion in the case when a different opinion value is observed twice, *R* is greater than 0.5, and no other opinions are observed — a behavior that does not align with reality but is presented here for comprehensiveness. We now explore varying responsiveness dependent on group identification under different model conditions.

At low exposure, *E* = 0.01, increasing tolerance tends to reduce polarization of the population. Furthermore, in the cases *T* = 0.1 and 0.4, around $R_{\text{in}}$ = 0.5 we can observe a smooth transition between higher polarization for greater $R_{\text{in}}$ and lower polarization for smaller $R_{\text{in}}$. We propose that this occurs due to the larger changes introduced by greater $R_{\text{in}}$, meaning that agents tend to react more strongly to the frequent exposures to in-groups, akin to increased noise in the system so it cannot settle.

At intermediary exposure, *E* = 0.05, the system presents the most variety of behaviors in the relation between $R_{\text{in}}$ and $R_{\text{out}}$ as we vary tolerance. Across tolerance levels, an $R_{\text{out}}$> 0.5 typically leads to polarization although this becomes less certain as tolerance increases. When considering $R_{\text{in}}$ we also see a tendency towards lower polarization when the parameter is lower for *T* = 0.01, it is unclear if this is also the case for T=0.1,0.4 since pixelation increases in these scenarios so the simulation outcomes are less clear. Accepting some exceptions increased responsiveness to both in-group and out-group typically results in increased polarization.

Finally, at high exposure, *E* = 0.25, almost all cases lead to maximum polarization no matter the level of group-dependent responsiveness. The exception being the case of low out-group responsiveness with high general tolerance ($R_{\text{out}}$< 0.2 and *T* = 0.4) which can result in lower polarization, this outcome arises due either to agents not overreacting to repulsive out-group or not overshooting attraction to in-group.

### Effects of Group-Dependent Exposure (GDE Simulations)

At low tolerance, the impact of increasing $E_{\text{out}}$ is to increase repulsion between groups as repulsive influences become a higher proportion. As tolerance increases, lower polarization becomes possible, although responsiveness also needs to remain at low levels. Note that the experiment under *R* = 0.01, *T* = 0.4 (low responsiveness, high tolerance) is the only experiment shown in Figs 4, 5, and 6, that arrives at complete consensus no matter what the group-dependent parameters are.

In-group exposure can be a tempering factor to reduce polarization when agents are not overly responsive. This occurs because the polarizing exposure to out-group opinions is countered by higher exposure to in-group opinions which result in an opinion update at each iteration that is dominated by movement towards in-group average.

Exposure to certain groups of users dependent on whether they are similar (in-group) or not (out-group) draws clear parallels with the much discussed topic of filter bubbles. Note however that the mechanism considered in our model integrates only a limited range of features that a Recommender System might include (namely homophily), notably excluding content-based recommendations or behavioral trace data (e.g., what users click, like, share). Yet, we observe that increasing the probability of observing out-group opinions by raising $E_{\text{out}}$ typically increases the level of polarization in the population. Similar to tolerance, the out-group parameter is more explanatory for the polarization behavior of the model; this backs up our understanding that polarizing behavior tends to occur through negative reaction to out-groups.

## Conclusion

In this paper, we showed how to integrate the notion of group identification into opinion dynamics models, which we illustrated using the Attraction Repulsion Model (ARM) as a way to improve analytical tools for studying polarization. Our choice was motivated by the need of a model that could produce polarized states in order for us to assess the role of group identification in polarization processes. In essence, our proposition — inspired by mainstream theoretical perspective in sociology and political sciences, but finding broader applications — is to include in models and simulations, at each iteration or time step, a phase in which agents assess the system to produce a partition of the whole populations into groups. This choice deliberately removes the persistence of group identification to focus on the interplay between groups and opinions. A necessary consequence of groupings is that each agent can identify every other member of the population as an in-group (if they belong to the same group) or as an out-group (if they do not). Our model then uses this partition to split parameters of exposure and influence into different values: one for in-groups and one for out-groups. Framing the evolution of opinions through this group-focused lens, we explored ranges of parameters for tolerated distances in opinion difference, responsiveness in opinion updates depending on the opinions of others, and exposure to other agents’ opinions.

We organized simulations for our model such that we were able to test the effect of group identification separately for the three main parameters of the opinion dynamics model on which we build our own: tolerance, responsiveness and exposure. If we compare across these three model parameters, we conclude that in most cases, tolerance of out-groups plays an important role to avoid complete polarization. This is relevant in connecting opinion dynamics simulations with more recent research linking *ideological* polarization (as measured, for instance, by the DER measure) and *affective* polarization or *animosity* towards out-groups [23,67,68]. In our model, affective polarization is integrated as a mechanism by allowing both the distinction of groups and the possibility of exerting comparatively negative (repulsive) influence among agents in different groups, while ideological polarization is reflected in the outcome of the distribution of positions of opinions of agents. Furthermore, our simulations show that exposure to the out-group should be limited unless tolerance is high and responsiveness is low, and high responsiveness to out-group should be minimised to evade highly polarized states in the systems covered by our setting. In particular, and even despite the simplifications incurred by our model, this observation is relevant to the debate surrounding the so-called filter bubbles and diversification as a remedy (i.e., exposing social media users to very diverse opinions). Our results highlight that backfires effects [36,51] should be considered in light of additional individual attributes such as those akin to tolerance in our model.

Typically, the final state of the populations’ opinions is determined by whether one group-dependent parameter is above or below a threshold. This is reflected by the vertical and horizontal boundaries separating zones of behavior in Figs 4, 5, and 6. Only under some of the group-dependent exposure (GDE) and one group-dependent responsiveness (GDR) simulations do we see different shapes of frontiers that suggest that a behavior change is due to a changing ratio between the in-group and out-group parameter. In other words, in these few instances the final state depends on the relation of parameter values to each other in a non-trivial fashion. However, in the broader parameter space, most outcomes rely on absolute values of parameters being within a certain range, so a single parameter separates behaviors between polarization and consensus.

In most experiments, the parameter modeling exposure, responsiveness and tolerance towards out-groups is the principal factor in determining whether the final state of the system is polarization or consensus. This showcases the importance of the notion of groups and *otherness* in social simulation, in accordance to theoretical expectations developed by social science research from which our model takes inspiration. Affective polarization (i.e., the expression of animosity towards out-groups, enabled in the model by including groups and parameters for influence across groups) is already considered as central to understanding ideological polarization (see for instance [23]), and the simulated behavior from the model concurs with that assessment, given that the majority of eventual system states are driven by out-group exposures.

This alignment between theoretical expectations and simulation results warrants the exploration of more complex models. In summary, this work strives to integrate one of the most salient features of social influence considered in social sciences and generally absent in opinion dynamics: group perception and identification. In integrating this feature in models and simulations, we have shown that it is of utmost importance, capable of dramatically modulating the transition between polarized and consensus states. This contribution points to several opportunities that may be explored to improve opinion dynamics models. In particular, we suggest to investigate different methods of implementing group identification in the opinion change process. For example, changing opinion after in-group or out-group exposures may be treated by different update functions. Secondly, different concepts of group identification could be explored, such as subjective perception of groupings where each individual perceives groups differently, to further close the gap between sociological theory and opinion dynamic models. For a different perspective on the results, the evolution of the exposure network could be investigated rather than focusing on the distribution of opinions. Exploration of more realistic models must address two simplifications of our model: 1) the lack of persistence of identity in time, and 2) additional and separate individual attributes on which identification may hinge, and that might be at interplay with opinion positions, or any other psychological construct that the model may target in an application. Finally, such a line of work should be fruitfully related to empirical data, to gain further insights on how these mechanisms unfold in real-life contexts. Recent empirical studies on social media have produced data on evolving groups within large online populations underpinned by political opinions. One study, for example, by Gaumont et al. [69], tracks the activity of 2.4M French Twitter (now X) users and infers their weekly group structure for the 44 weeks leading to the 2017 French presidential elections by inspecting activity and probable exposure. A related study investigates how individuals from these groups engage in hostile communication with out-groups [70]. Our model, through the explicit analytical incorporation of group structures, affords additional methodological avenues for bridging observational data and opinion dynamics models. When data pertaining groups is available, our model offers the possibility of informing priors for group structures, thus enabling additional contact between models and empirical data.

## Supporting information

S1 File Supporting information document. Document with additional content contextualizing figures S1, S2, S3, and S4 Figs.
(PDF)

S1 FigChanging the Starting Population Distribution.Group-Dependent Tolerance (GDT) simulations *under different starting distributions for the population*. In the ‘unimodal’ case, the agents’ opinions are distributed by a single Normal distribution $𝒩(\mu_{1},\sigma_{1}^{2})$ with $\mu_{1}=0.5,\sigma_{1}=0.05$; in the ‘wider bimodal’ case the population follows two distributions as is described in the Results with $\mu_{1}=0.2,\mu_{2}=0.8$ while $\sigma_{1}=\sigma_{2}=0.05$. The most important factor in determining polarization outcomes of the population remains $T_{\text{out}}$.(PDF)

S2 Fig Convergence methodology. Evolution of DER polarization in experiments under various parameter combinations. Stability is typically reached by 300 iterations for high-, mid-, and no-, polarization. The curves correspond to the same example polarization behaviors found for the conditions in Fig 3.
(PNG)

S3 FigStatic groups.These plots follow the same experimental procedure as in the main body of the article except that groups are not updated at each time iteration; they are defined at the start of the simulation and never updated, so we term them as ‘static’. They should be compared to the middle plot of Fig 4, as well as Fig 3d. In the case of (a) above there is only high polarization when $T_{\text{out}}$ < 0.25 and $T_{\text{in}}$ > 0.25 while in the standard article case lower polarization occurs—this is because splinter groups are pulled back to their original groups in the static case (when $T_{\text{in}}$ is sufficient) since both sides continue to consider the other as in-group and so no middle ground groups can establish. The lack of any new groups can be seen in (b) above which would produce fragmentation of groups with the given parameters under the updating groups case.(PDF)

S4 FigDuclos-Esteban-Ray polarization measure.Computation of the Duclos-Esteban-Ray (DER) polarisation measure used on synthetic data drawn from a 2-component Gaussian Mixture Model with a parameter α=0.5 as used in the article.(PDF)
