# Supplementary material for: Co-evolution of groups and opinions in an agent-based model
Source: PLoS One. 2025 Dec 12;20(12):e0338486. doi: 10.1371/journal.pone.0338486 (PMC12700461; doi:10.1371/journal.pone.0338486)
Supplement: S1 File — Document with additional content contextualizing figures S1, S2, S3, and S4 Figs. (PDF) [file pone.0338486.s001.pdf]

Supporting Information for Co-evolution of groups and opinions in agent-based models

Duncan Cassells, Antoine Vendeville, Lionel Tabourier, Pedro Ramaciotti

.

S1: Changing the Starting Population Distribution

The plots in Fig. S1 are for the case where $R = 0.1, E = 0.1$ (as in the central sub-plot of Fig. 4) except that the starting distribution of opinions is changed at $t = 0$. In the case of the 'Unimodal' plot, the population follows a single Normal distribution $\mathcal{N}(\mu_1, \sigma_1^2)$ with $\mu_1 = 0.5, \sigma_1 = 0.05$. While the 'Wider Bimodal' case presents our usual case of drawing from two Normal distributions but we set $\mu_1 = 0.2, \mu_2 = 0.8$ while maintaining the original standard deviation $\sigma_1 = \sigma_2 = 0.05$, therefore the two initial opinion groups begin the experiments with a larger difference in opinion than our standard case presented in the paper.

These results with differing initial distributions create heatmaps that are approximately equivalent to what is observed in Fig. 4. That is not to say that the results are the same but that exact parameter thresholds are dependent on the starting distribution while general behavior is unchanged. It is true that the point at which T_{out} changes population outcomes from high polarization to low polarization is different as a result of different starting opinion distributions, and that high polarization outcomes are generally less likely in the unimodal case. However, in each case the distinct zones of behavior continue to exist, have equal DER polarization values, and have consistent relations to each other. Therefore despite different starting conditions, for the case where $E = 0.1, R = 0.1$, the results present macro-level behavior of model parameters that is consistent across simulations studied here.

S2: Convergence Methodology

Fig. S2 shows convergence to the final DER values that allow us to discuss polarization outcomes of the experiments in Fig. 4, 5 and 6. Final values are either constant or approximately so, our criterion is that the average polarization for an experiment should change less than 2% in the final one hundred iterations to be considered approximately constant.

As mentioned in the main body of the text, our simulations terminate after 1000 iterations or if there is no model change for 100 iterations. After at most 300 iterations, the simulations are stable at either constant values, or values with small variation in the case of 'unstable polarization'. This gives us the necessary confidence to be able to discuss the polarization outcome of the system across parameter combinations.

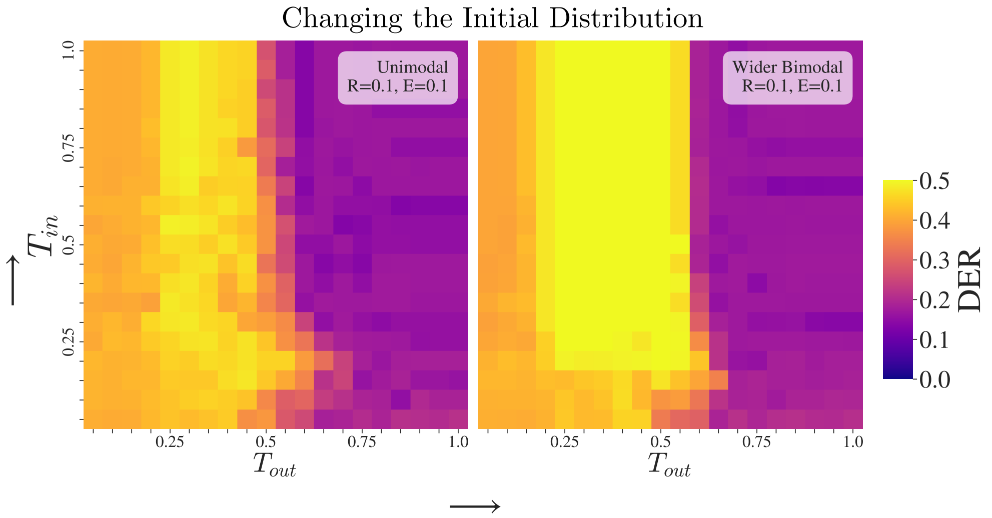

Figure S1: Group-Dependent Tolerance (GDT) simulations *under different starting distributions for the population.* In the 'unimodal' case, the agents' opinions are distributed by a single Normal distribution $\mathcal{N}(\mu_1, \sigma_1^2)$ with $\mu_1 = 0.5, \sigma_1 = 0.05$; in the 'wider bimodal' case the population follows two distributions as is described in the Results with $\mu_1 = 0.2, \mu_2 = 0.8$ while $\sigma_1 = \sigma_2 = 0.05$. The most important factor in determining polarization outcomes of the population remains T_{out} .

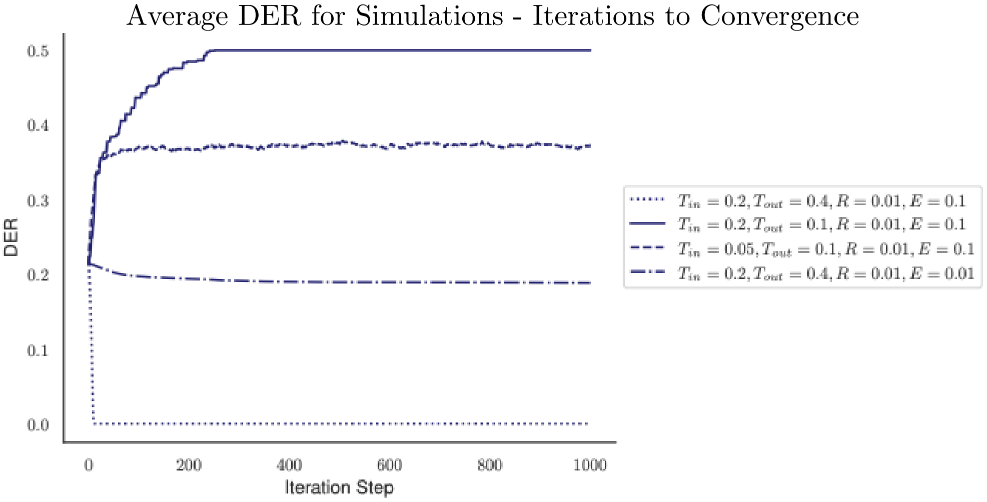

Figure S2: Evolution of DER polarization in experiments under various parameter combinations. Stability is typically reached by 300 iterations for high-, mid-, and no-, polarization. The curves correspond to the same example polarization behaviors found for the conditions in Fig. 3.

S3: Duclos-Esteban-Ray polarization measure

In this section we provide computations of the Duclos-Esteban-Ray (DER) polarization measure used on synthetic data drawn from a 2-component Gaussian Mixture Model to illustrate how the DER measure captures two key properties assured by the axiomatic construction of the measure: distance between modes and spread around each mode.

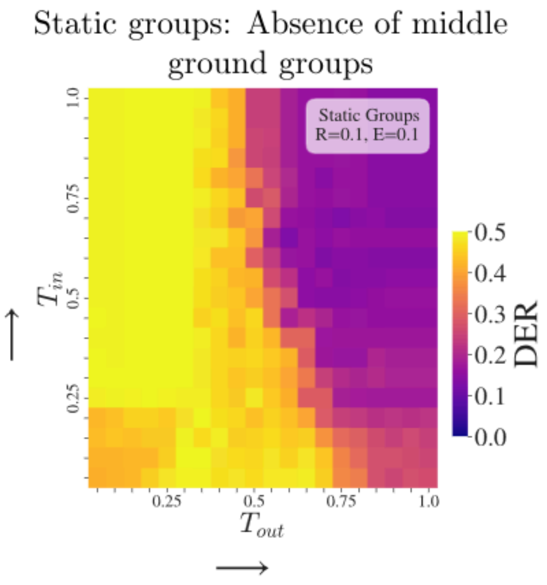

(a) $T_{\text{in}} = 0.5$, $T_{\text{out}} = 0.4$, $R = 0.01$, $E = 0.1$

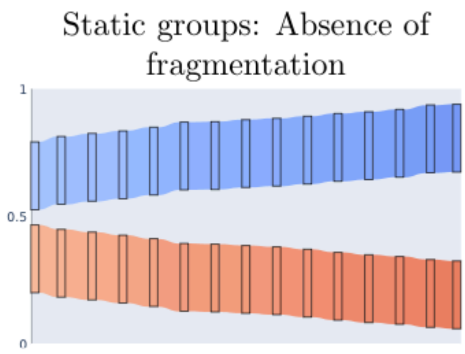

(b) $T_{\text{in}} = 0.05$, $T_{\text{out}} = 0.1$, $R = 0.01$, $E = 0.1$

Figure S3: These plots follow the same experimental procedure as in the main body of the article except that groups are not updated at each time iteration; they are defined at the start of the simulation and never updated, so we term them as 'static'. They should be compared to the middle plot of Fig. 4, as well as Fig. 3d. In the case of (a) above there is only high polarization when $T_{\text{out}} < 0.25$ and $T_{\text{in}} > 0.25$ while in the standard article case lower polarization occurs – this is because splinter groups are pulled back to their original groups in the static case (when T_{in} is sufficient) since both sides continue to consider the other as in-group and so no middle ground groups can establish. The lack of any new groups can be seen in (b) above which would produce fragmentation of groups with the given parameters under the updating groups case.

$$f = Gaussian(\mu_1, \sigma_1) + Gaussian(\mu_2, \sigma_2) \tag{1}$$

$$P_\alpha(f) \equiv \int\int f(x)^{1+\alpha} f(y)|y-x|dydx. \tag{2}$$

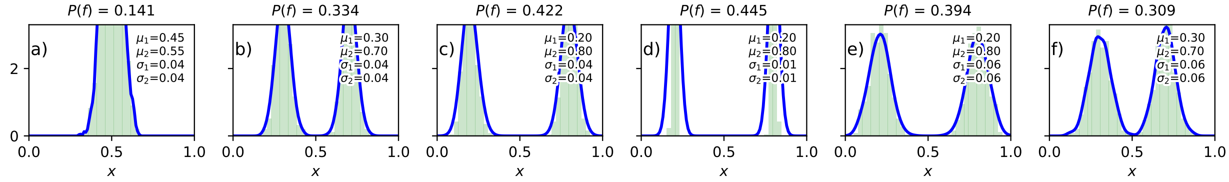

Figure S4: Computation of the Duclos-Esteban-Ray (DER) polarisation measure used on synthetic data drawn from a 2-component Gaussian Mixture Model with a parameter $\alpha = 0.5$ as used in the article.
