# Supplementary figures and images for: Co-evolution of groups and opinions in an agent-based model
Source: PLoS One. 2025 Dec 12;20(12):e0338486. doi: 10.1371/journal.pone.0338486 (PMC12700461; doi:10.1371/journal.pone.0338486)

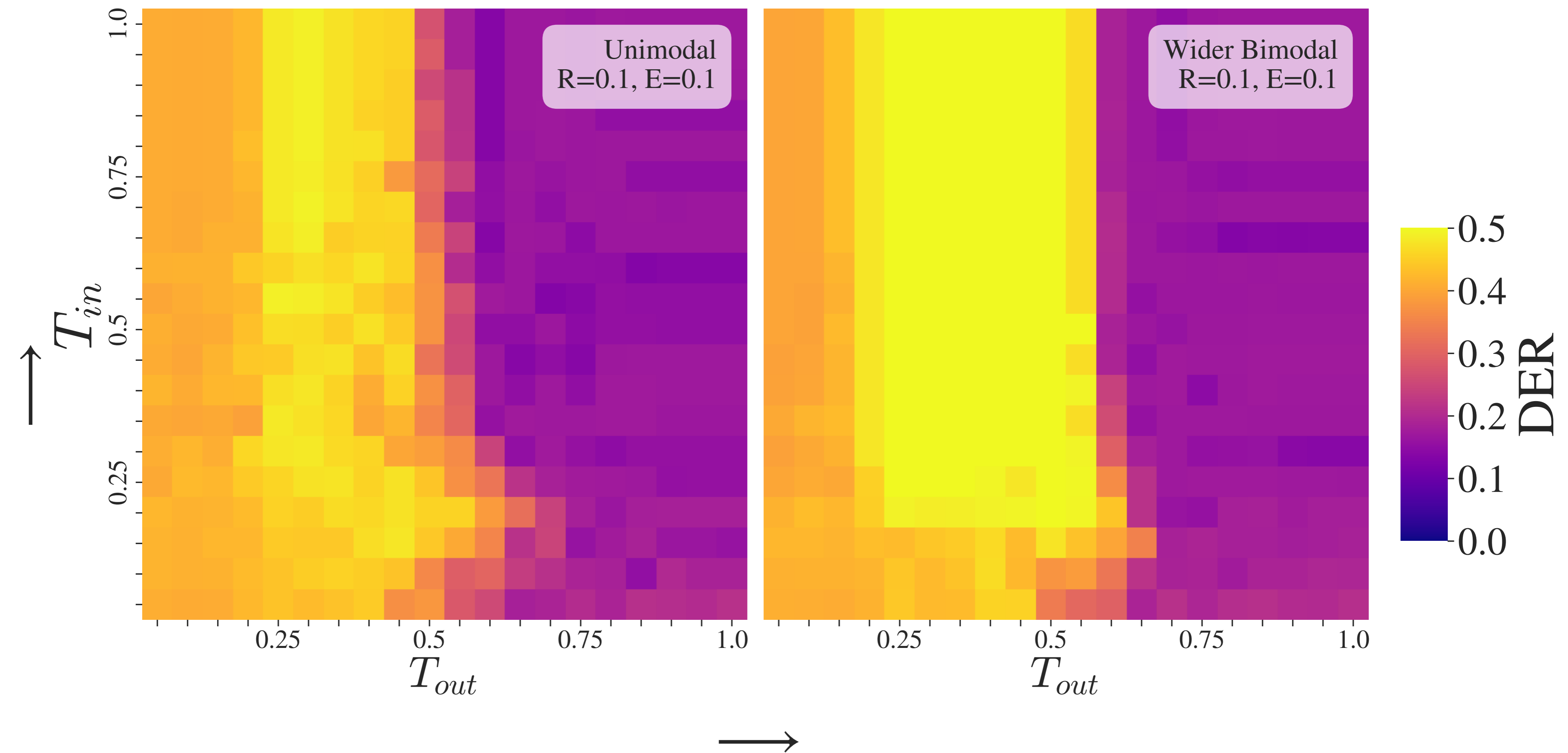

Supplement: S1 Fig — Group-Dependent Tolerance (GDT) simulations under different starting distributions for the population. In the ‘unimodal’ case, the agents’ opinions are distributed by a single Normal distribution 𝒩(μ1,σ12) with μ1=0.5,σ1=0.05; in the ‘wider bimodal’ case the population follows two distributions as is described in the Results with μ1=0.2,μ2=0.8 while σ1=σ2=0.05. The most important factor in determining polarization outcomes of the population remains Tout. (PDF) [file pone.0338486.s002.pdf]

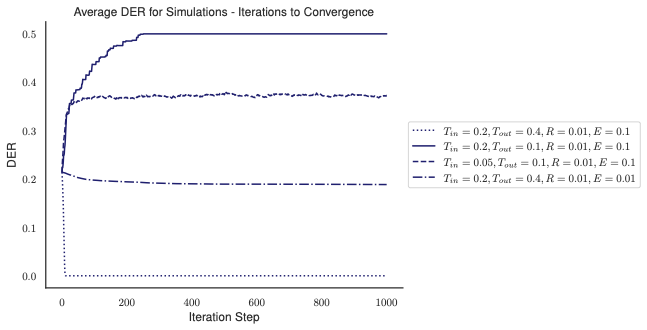

Supplement: S2 Fig — Evolution of DER polarization in experiments under various parameter combinations. Stability is typically reached by 300 iterations for high-, mid-, and no-, polarization. The curves correspond to the same example polarization behaviors found for the conditions in Fig 3. (PNG) [file pone.0338486.s003.png]

$$P(f) = 0.141$$

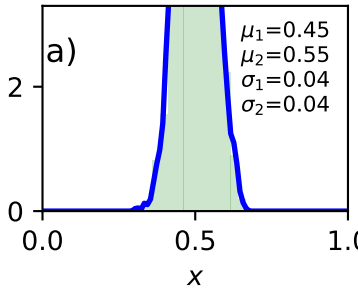

$$P(f) = 0.334$$

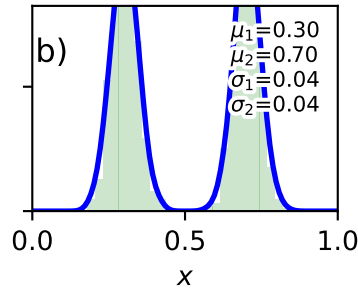

$$P(f) = 0.422$$

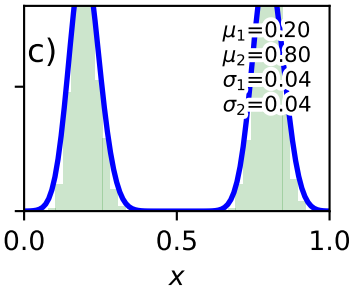

$$P(f) = 0.445$$

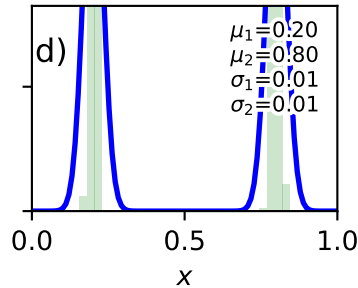

$$P(f) = 0.394$$

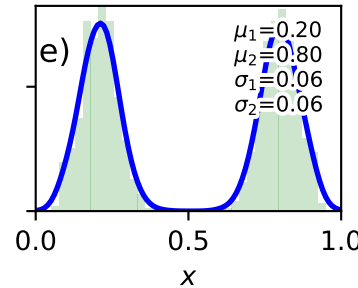

$$P(f) = 0.309$$

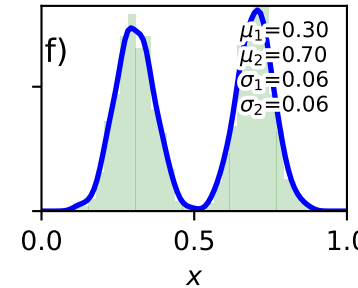

Supplement: S4 Fig — Computation of the Duclos-Esteban-Ray (DER) polarisation measure used on synthetic data drawn from a 2-component Gaussian Mixture Model with a parameter α=0.5 as used in the article. (PDF) [file pone.0338486.s005.pdf]
