# Supplementary material for: Co-evolution of groups and opinions in an agent-based model
Source: PLoS One. 2025 Dec 12;20(12):e0338486. doi: 10.1371/journal.pone.0338486 (PMC12700461; doi:10.1371/journal.pone.0338486)
Supplement: S3 Fig — These plots follow the same experimental procedure as in the main body of the article except that groups are not updated at each time iteration; they are defined at the start of the simulation and never updated, so we term them as ‘static’. They should be compared to the middle plot of Fig 4, as well as Fig 3d. In the case of (a) above there is only high polarization when Tout < 0.25 and Tin > 0.25 while in the standard article case lower polarization occurs—this is because splinter groups are pulled back to their original groups in the static case (when Tin is sufficient) since both sides continue to consider the other as in-group and so no middle ground groups can establish. The lack of any new groups can be seen in (b) above which would produce fragmentation of groups with the given parameters under the updating groups case. (PDF) [file pone.0338486.s004.pdf]

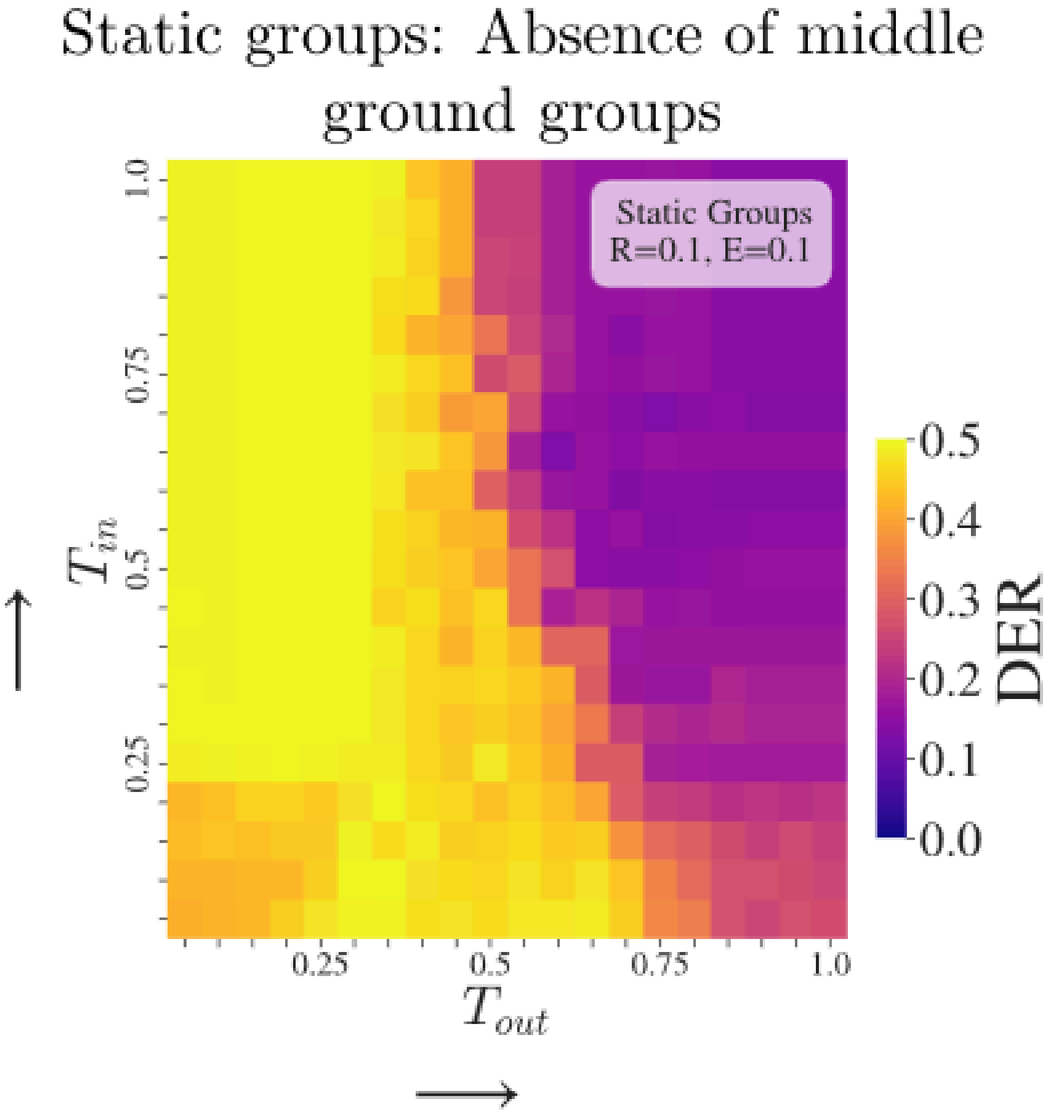

(a) $T_{\text{in}} = 0.5$, $T_{\text{out}} = 0.4$, $R = 0.01$, $E = 0.1$

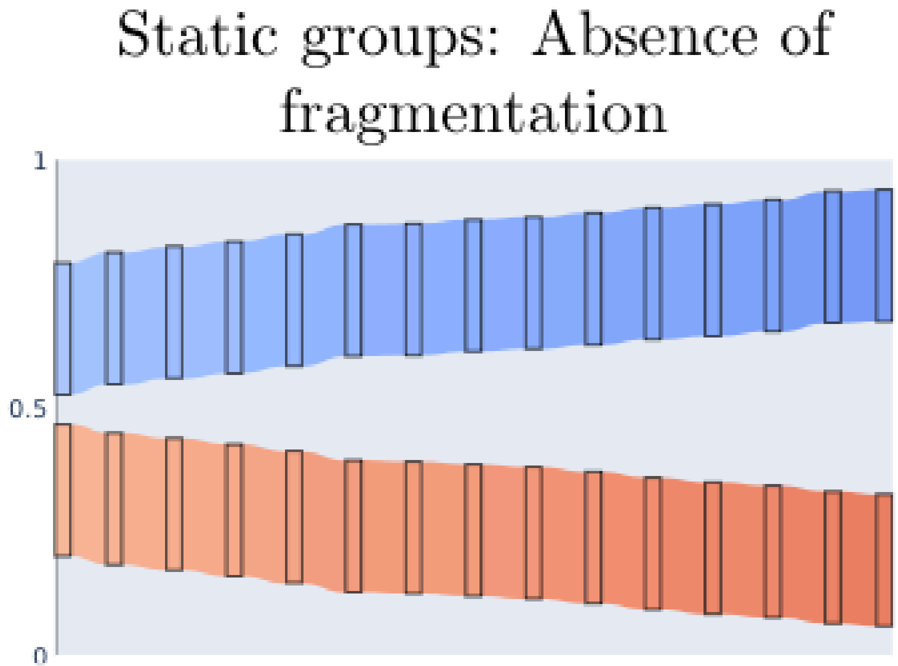

(b) $T_{\text{in}} = 0.05$, $T_{\text{out}} = 0.1$, $R = 0.01$, $E = 0.1$
